# *In silico* Exploration of Inhibitors for SARS-CoV-2's Papain-Like Protease

**DOI:** 10.3389/fchem.2020.624163

**Published:** 2021-02-04

**Authors:** Tien Huynh, Wendy Cornell, Binquan Luan

**Affiliations:** Computational Biological Center, IBM Thomas J. Watson Research, New York, NY, United States

**Keywords:** COVID-19, SARS-CoV-2, papain-like protease, docking, molecular dynamics simulation (MD)

## Abstract

Coronavirus disease 2019 (COVID-19) is an ongoing global pandemic caused by severe acute respiratory syndrome coronavirus 2 (SARS-CoV-2), with very limited treatments so far. Demonstrated with good druggability, two major proteases of SARS-CoV-2, namely main protease (Mpro) and papain-like protease (PLpro) that are essential for viral maturation, have become the targets for many newly designed inhibitors. Unlike Mpro that has been heavily investigated, PLpro is not well-studied so far. Here, we carried out the *in silico* high-throughput screening of all FDA-approved drugs *via* the flexible docking simulation for potential inhibitors of PLpro and explored the molecular mechanism of binding between a known inhibitor rac5c and PLpro. Our results, from molecular dynamics simulation, show that the chances of drug repurposing for PLpro might be low. On the other hand, our long (about 450 ns) MD simulation confirms that rac5c can be bound stably inside the substrate-binding site of PLpro and unveils the molecular mechanism of binding for the rac5c-PLpro complex. The latter may help perform further structural optimization and design potent leads for inhibiting PLpro.

**Graphical Abstract:**
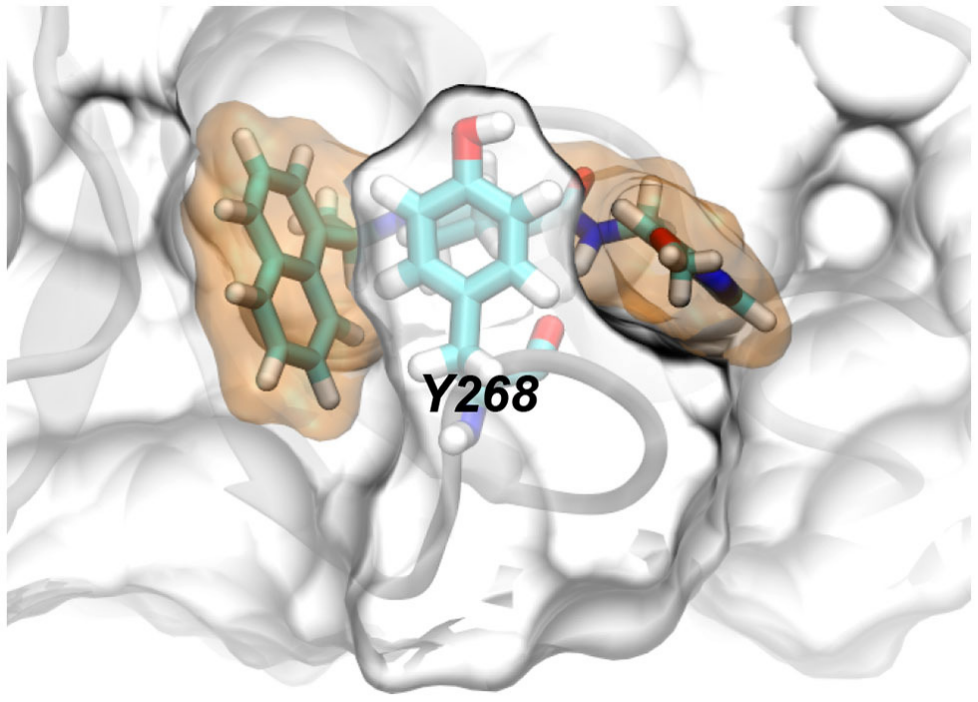
Y268 in papain-like protease coordinates the binding of rac5c.

## 1. Introduction

The ongoing COVID-19 pandemic, which emerged in China at the end of 2019, is caused by the third severely pathogenic novel coronavirus since the beginning of the twenty-first century. This new virus known as severe acute respiratory syndrome coronavirus 2 (SARS-CoV-2) is similar to, yet distinct from, the previous two zoonotic coronaviruses, SARS-CoV and MERS-CoV, which also caused serious and atypical pneumonia (Kuiken et al., 2003; Bermingham et al., 2012; Wu et al., 2020). COVID-19 is a highly contagious disease that can spread very easily between people through liquid droplets (or aerosols) which come out of infected persons when they sneeze, cough, or even talk. People with COVID-19 can spread the virus before they show any symptoms or realize that they are sick, and become especially infectious when they start showing symptoms, including fever, chills, cough, sore throat, congestion, shortness of breath, headache, muscle pain, diarrhea, vomiting, and loss of smell or taste. Most COVID-19 infected individuals will only have mild symptoms (or no symptoms at all). However, those with certain underlying health conditions (such as cardiovascular disease, chronic respiratory disease, or diabetes) can become severely sick with fatal outcomes. So far, the COVID-19 pandemic has unleashed an unprecedented crisis in a very short period of time. Beyond the spread of the disease itself, which has resulted in over 39 million laboratory-confirmed cases of infection globally with over 1 million reported deaths as of October 17, 2020 (https://covid19.who.int), efforts to quarantine it have caused far-reaching impact on human physical well-being in societies, on economic development and prosperity, as well as on political systems around the globe. As the entire world battles this pandemic, scientists and researchers are racing against time to search for a vaccine, drug, or therapy to get rid of the novel coronavirus. Despite all of these efforts, it is believed that the COVID-19 pandemic might not be under full control anytime soon and might play out until next year and beyond (Scudellari, 2020).

Currently, many existing drugs (including remdesivir) have been tested or are being tested in clinics to expedite the discovery of drugs for treating COVID-19 patients. With a known safety profile and bioavailability, an existing FDA-approved drug which shows a strong inhibitory effect on key targets (such as the viral proteases or RNA-dependent RNA polymerase) of SARS-CoV-2 could be quickly repurposed for COVID-19. For example, remdesivir which can inhibit the biological function of RNA-dependent RNA polymerase of SARS-CoV-2 was shown to shorten the hospital stay (Beigel et al., 2020) and was recently approved by the FDA. Meanwhile, papain-like protease (PLpro) and 3-chymotrypsin-like protease (3CLPro or Mpro) are proteins encoded in the SARS-CoV-2 genome that are known to play an important role in the viral replication process and hence are attractive antiviral drug targets to fight against COVID-19 (Luan et al., 2020). So far, Mpro has been extensively investigated to identify potent inhibitors (Huynh et al., 2020a,b; Jin et al., 2020; Zhang et al., 2020), however PLpro was much less studied mainly due to the lack of crystal structures. Previous studies (Barretto et al., 2005; Ratia et al., 2008; Báez-Santos et al., 2015; Shin et al., 2020) on SARS-CoV showed that besides working together with Mpro to process the 16 non-structural proteins (nsps) for assembly of the viral replicase complex to initiate the replication and transcription of the viral genome, PLpro also has significant functional implications on the host innate immune responses. Therefore, it is warranted to search for antiviral drugs to target PLpro, inhibiting not only the viral replication but also the dysregulation of signaling cascades in infected cells to protect the surrounding healthy ones.

**Figure 1:**
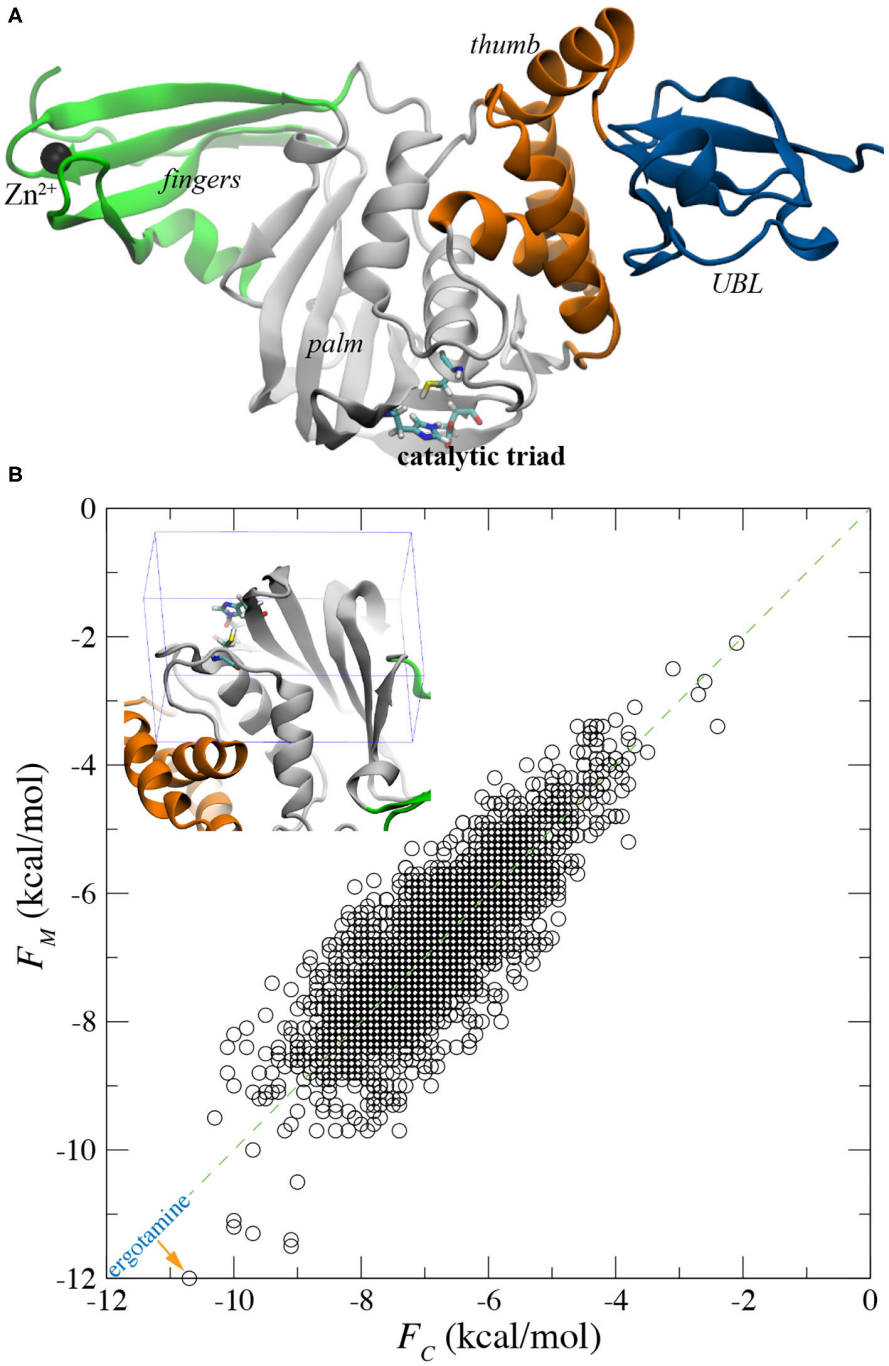
Flexible docking for screening all FDA-approved drugs for binding with PLpro. **(A)** Illustration of PLpro in the cartoon representation. The entire protein contains the ubiquitin-like (UBL), thumb, palm, and fingers domains that are colored in blue, orange, gray and green, respectively. In the active site, the catalytic triad C111-H272-D286 is in the stick representation. The bound Zn^2+^ ion in the fingers domain is shown as a black sphere. **(B)** A scatter plot showing affinity scores *F*_*M*_ and *F*_*C*_ for all FDA approved drugs docked in the active sites of the MD-equilibrated PLpro structure and the crystal PLpro structure, respectively. *Inset*: an illustration of the box around the active site within which all drug molecules were docked.

Recently, a number of crystal structures (Gao et al., 2020; Rut et al., 2020; Shin et al., 2020) for SARS-CoV-2's PLpro became available, which helps unveil its structure-related activities. Similar to PLpro of previous SARS-CoV, PLpro of emerging SARS-CoV-2 contains four domains linked with flexible cords. Namely, fingers domain, palm domain, thumb domain and Ubiquitin-like (UBL) domain (Figure 1). Notably, there is a bound Zn^2+^ ion in the fingers domain and a catalytic triad (in the active site) in the palm domain. Near the catalytic triad, there exists a groove-like pocket that can harbor a protein ligand and possibly guide the ligand toward the triad for cleavage. For example, the C-terminal region of the protein mISG15 can be bound inside the pocket (PDB entry: 6YVA) (Shin et al., 2020). Due to the good druggability, recent research efforts have been focused on the discovery of inhibitors targeting this substrate binding pocket. So far, some covalently bound peptide ligands [such as VIR250 and VIR251, Rut et al., 2020] as well as small molecules [such as GRL-0617 (Gao et al., 2020; Shin et al., 2020) and rac5c (Klemm et al., 2020)] have been found to occupy the pocket, inhibiting the function of PLpro.

The combination of experimental and computational approaches is an important strategy for the discovery of novel and promising compounds. Molecular docking is a powerful method to predict the conformations of ligands within a macromolecule's substrate-binding site and roughly estimate the corresponding receptor-ligand binding affinity, which is usually applied at the initial stage of structure-based drug design process. While this paper was in the process of review, two docking-based *in silico* studies for repurposing existing drugs to inhibit SARS-CoV-2's PLpro were published (Delre et al., 2020; Ibrahim et al., 2020). Additionally, molecular dynamics (MD) simulations have been shown to yield predictions [such as the binding affinity for a ligand-protein complex Huynh et al., 2020a; Luan and Huynh, 2020] consistent with experimental results, with the ever-improving force fields. Motivated to find promising leads for SARS-CoV-2's PLpro, we carried out *in silico* modeling of all FDA-approved drugs inside the substrate-binding pocket for potential drug repurposing and also investigated the molecular mechanism of a known inhibitor rac5c bound in the same pocket (no crystal structure yet). Thus, this work provides insights derived from the computational studies intended to inform discovery and optimization of lead molecules and to manage expectations for the potential to repurpose approved drugs against this relatively new and less well-studied target.

## 2. Results and Discussions

### 2.1. High-Throughput Screening of All FDA Approved Drugs

For the docking study, we first obtained the atomic (or crystal) structure of SARS-CoV-2's PLpro from the protein data bank (PDB) with the entry code 6WX4, after removing its covalently bound ligand (VIR251). The apo structure of PLpro was either directly utilized in the docking study or was further equilibrated in a 0.15 M NaCl electrolyte (see Supplementary Figure 1A) using the MD simulation before its use in the docking study. After about 50 ns of MD simulation, the PLpro's structure was properly equilibrated in the physiology-relevant environment as evidenced by root-mean-square-deviation (RMSD) values of the protein backbone (Supplementary Figure 1B). Due to the presence of multiple domains in flexible PLpro (Figure 1A), RMSD for the backbone of entire PLpro fluctuated between 1.5 and 3.5 Å during the rest of 150 ns of simulation (Supplementary Figure 1B). However, for the backbone in the palm domain (harboring the substrate-binding site), RMSD values saturated at about 1.3 Å (Supplementary Figure 1B), indicating that the palm domain is structurally stable. During the simulation, we observed that the Zn^2^+ ions were stably coordinated with four deprotonated cysteins (C189, C192, C224, and C226) in the fingers domain (see Supplementary Video 2).

**Figure 2:**
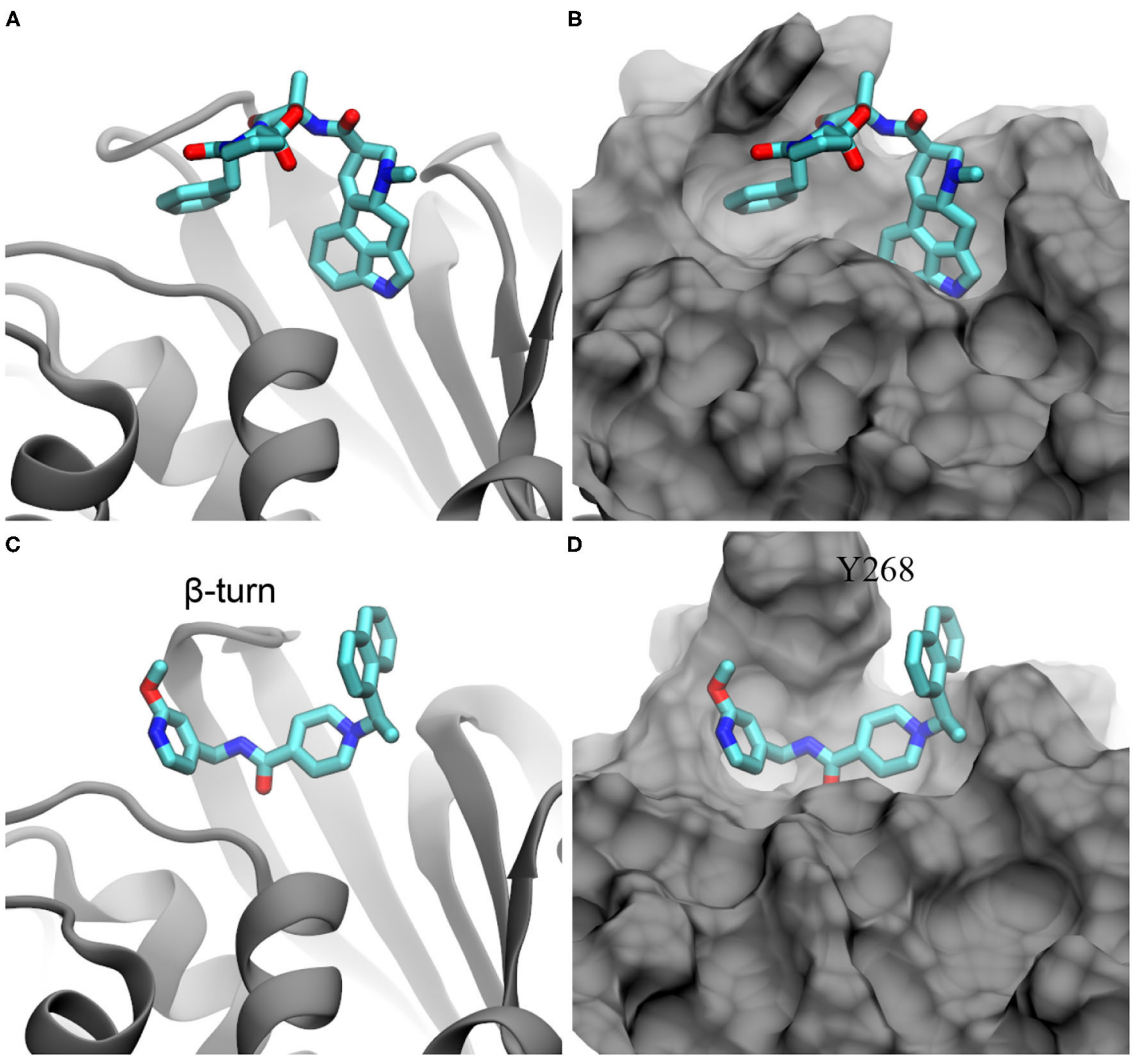
Illustration of ergotamine and rac5c (in stick representation) inside the substrate-binding site of PLpro. **(A,B)** The docked pose of ergotamine inside the PLpro's active site. **(C,D)** The hypothesized pose of rac5c inside the PLpro's active site, according to rac3j in the substrate-binding site of PLpro from SARS-CoV (PDB entry: 4OVZ). PLpro is in the cartoon representation in **(A,C)** and is in the molecular surface representation in **(B,D)**.

With the crystal and MD-equilibrated structures of PLpro, we performed flexible-docking-based screening of the entire FDA-approved drug library (see section 4 for details). The inset of Figure 1B shows the pocket-containing simulation box within which conformations of each drug were searched for. Employing 22 IBM power nodes (with twenty 8-way cores in each node), the docking of all FDA-approved drugs in each PLpro structure took about 22 h, compared with 45 min for the rigid docking using same modeling parameters (see section 4). With the binding affinities *F*_*M*_ and *F*_*C*_ for drugs docked in MD-equilibrated and crystal structures respectively, we highlight the docking results in a scatter plot shown in Figure 1B. Two sets of data (*F*_*M*_ and *F*_*C*_) appear to be well-correlated, with a majority of data falling on the line defined by *y* = *x* in the plot. The broadening of data indicates the variation of docking scores for two PLpro structures. Remarkably, with the flexible docking enabled, we found the same top candidate (ergotamine) for both PLpro structures, with the affinity scores −12.0 and −10.7 kcal/mol for the MD-equilibrated and crystal structures, respectively. The best conformation of ergotamine in the MD-equilibrated PLpro is illustrated in Figures 2A,B, which highlights that the hydrophobic interaction is dominant. Furthermore, we carried out MD simulation to evaluate the stability of ergotamine inside the pocket (see below).

It is worth mentioning that when applying the rigid docking approach outcomes can be different for two PLpro structures. Overall, more data points in the scatter plot lie above the *y* = *x* line (Supplementary Figure 2), suggesting that generally *F*_*M*_ is larger than *F*_*C*_. The correlation coefficient *R*^2^ for rigid-docking results is 0.58, which is less than the *R*^2^ value (0.66) for the flexible docking results (Figure 1B). Overall, results from the flexible docking are less dependent on the different PLpro structure than the ones from rigid docking. Additionally, these affinity scores from the rigid docking (Supplementary Figure 2) are about 2–3 kcal/mol larger (i.e., weaker binding) than those obtained from the flexible docking. The rigid docking yielded two different top candidates for the two PLpro structures, with aprepitant (−9.2 kcal/mol) for the crystal structure and lomitapide (−8.7 kcal/mol) for the MD-equilibrated structure.

### 2.2. Molecular Dynamics Simulations for Ergotamine and rac5c in PLpro' Pocket

Recently, it was demonstrated in an experiment that rac5c, a known inhibitor of SARS-CoV's PLpro, can also inhibit SARS-CoV-2's PLpro with an IC50 value of 0.81 μM (Klemm et al., 2020). So far, the crystal structure for the rac5c-PLpro complex is still not available, which hinders the understanding of its binding mechanism. Previously, rac3j (with a molecular structure similar to the one of rac5c) was co-crystallized with SARS-CoV's PLpro (Báez-Santos et al., 2014). However, it is noted that in that crystal environment PLpro forms a dimer structure (Supplementary Figure 3) where two substrate-binding sites in the PLpro dimer are close to each other. Consequently, two rac3j ligands in their respective PLpro's pockets are in contact with each other and thus the rac3j's conformation can be affected by the proximity of a neighboring copy in the crystal structure. Additionally, each PLpro's pocket is occupied not only by a rac3j ligand but also a DMSO molecule (a co-crystallization agent), as shown in Supplementary Figure 3. It is not clear whether a rac3j ligand is stabilized due to the presence of the DMSO molecule. To reveal the molecular mechanism for rac5c's binding with PLpro, we carried out MD simulation for the rac5c-PLpro complex in a 0.15 M NaCl electrolyte. The initial complex structure before equilibration in MD simulation is shown in Figures 2C,D, built according to the rac3j's conformation in the SARS-CoV's PLpro. It is noted that rac5c is the R-enantiomer for the best fit inside the pocket (Báez-Santos et al., 2014). In addition, ergotamine discovered from the flexible docking occupies a slightly different location inside the pocket, from the one occupied by rac5c in the pocket of PLpro (Figure 2).

**Figure 3:**
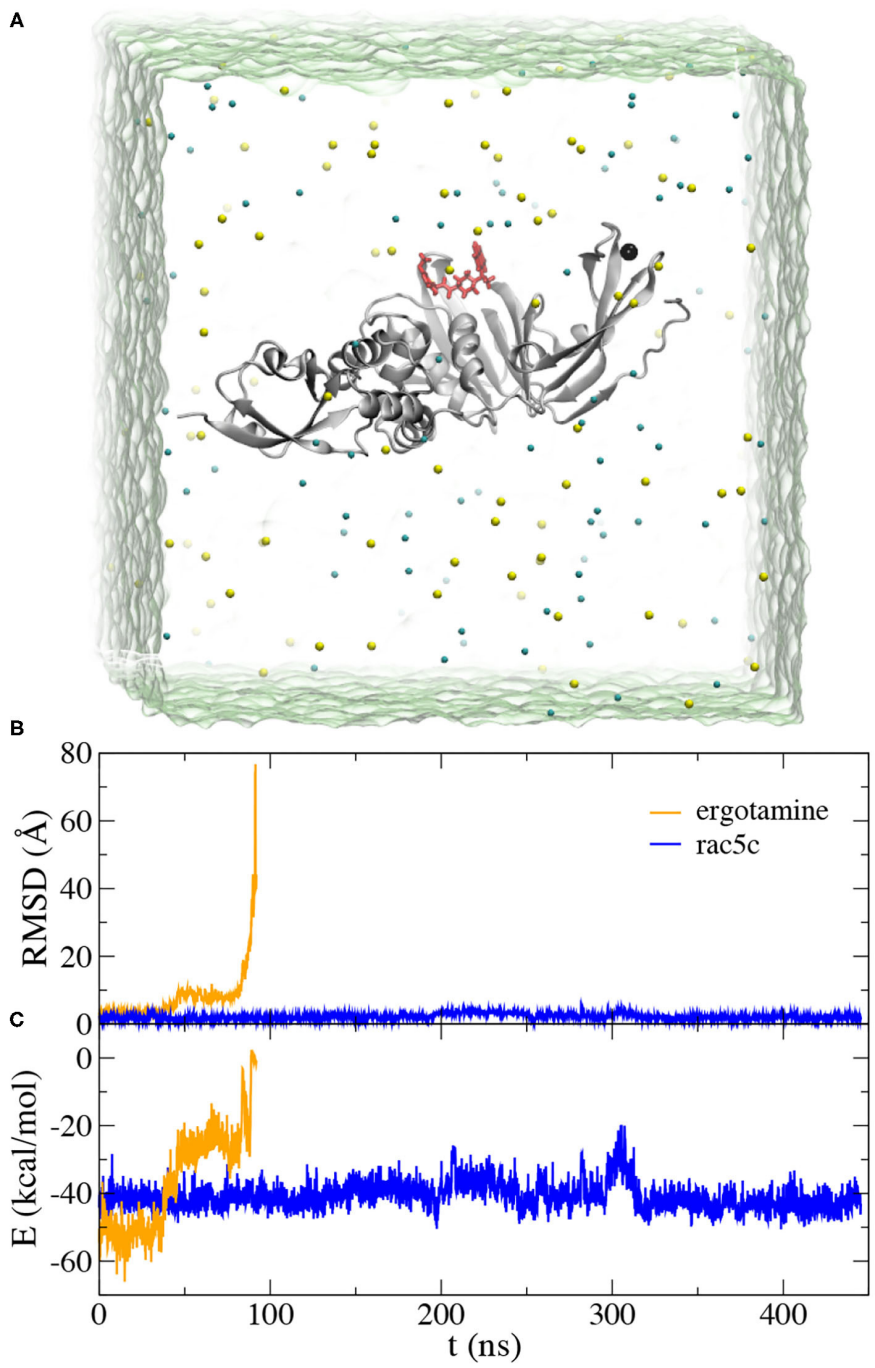
MD simulation for ergotamine and rac5c inside the active site of PLpro. **(A)** Illustration of MD simulation system. PLpro (gray) is in the cartoon representation and the bound ligand (red) is in the stick representation. Na^+^ and Cl^−^ ions are shown as yellow and cyan spheres, respectively. Water (green) is transparent. **(B)** Root-mean-square-deviation (RMSD) values for the bound ligands, ergotamine (orange) and rac5c (blue), when the PLpro's backbones in the simulation trajectory were aligned. **(C)** Interaction energies *E* (including van der Waals and electrostatic ones) between the ligand (ergotamine or rac5c) and PLpro calculated from simulation trajectories.

Following our MD simulation protocol (see section 4) that was calibrated to investigate the binding between drug molecules and SARS-CoV-2's Mpro (Huynh et al., 2020a), we performed all-atom MD simulations to investigate the stability of ergotamine and rac5c inside the PLpro's substrate-binding pocket. Figure 3A illustrates the simulation system that PLpro with a bound inhibitor (either ergotamine or rac5c) was solvated in a 0.15 M NaCl electrolyte.

During the MD simulation of the ergotamine-PLpro complex (see Movie PLpro-ergotamine.mpg), we observed that ergotamine was only stable inside the pocket for about 40 ns. During this time, ergotamine formed hydrophobic interactions with Y264, P247, P248, and M208, mainly through its functional group 4,6,6a,7,8,9-hexahydroindolo[4,3-fg]quinoline. The benzene ring on the other end of molecule only weakly interacted with the hydrophobic residue L162. From the trajectory analysis, after aligning all PLpro according to their backbone structures, we calculated RMSD values for ergotamine (non-hydrogen atoms) against its starting conformation. Consistently, the RMSD values remained at 3.5 Å for the first 40 ns of MD simulation (Figure 3B). After that, the benzene ring started drifting away from its original position, while the fragment 4,6,6a,7,8,9-hexahydroindolo[4,3-fg]quinoline remained to be stably bound inside the pocket. In this stage (from 40 to 80 ns), the average RMSD value is about 8.0 Å (Figure 3B). During the remaining MD simulation, ergotamine gradually diffused away from the pocket as reflected by large RMSD values (Figure 3B).

Thus, despite the good docking score (−12.0 kcal/mol), we found that in the physiology-like environment ergotamine was not stable inside the PLpro's pocket. We also carried out MD simulation for aprepitant (the best candidate from the rigid docking study) in the PLpro's pocket and it was not stably bound too (diffusing away in a few nanoseconds). From these MD simulations, it is premature to state that the chance of drug repurposing for PLpro is little, however in recent high-throughput experimental screening (Klemm et al., 2020) it was concluded that the repurposing of existing drug molecules is unlikely to yield drug candidates for PLpro. Therefore, our simulation results are somewhat consistent with the conclusion from the experiment and it might be more promising by exploring/optimizing other known inhibitors for PLpro.

As expected, in MD simulation of the rac5c-PLpro complex we found that rac5c was stably bound inside the PLpro's pocket for the entire ~450 ns simulation (see Movie PLpro-rac5c.mpg). Notably, RMSD values for rac5c kept at about 1.5 Å, corroborating its stable binding inside the PLpro's pocket. Additionally, we calculated interaction energies including van der Waals and electrostatic (dielectric constant~4) ones between rac5c and PLpro (Figure 3B). Overall, interaction energies are nearly constant (indicating stable binding) except for one instability event occurring around 300 ns (see more discussion below). For comparison, interaction energies between ergotamine and PLpro quickly increased to about zero (i.e., no interaction) when ergotamine escaped from the PLpro's pocket (Figure 3C). Therefore, our simulation results are consistent with experimental ones showing that rac5c can be an efficacious inhibitor for PLpro (Klemm et al., 2020).

**Figure 4:**
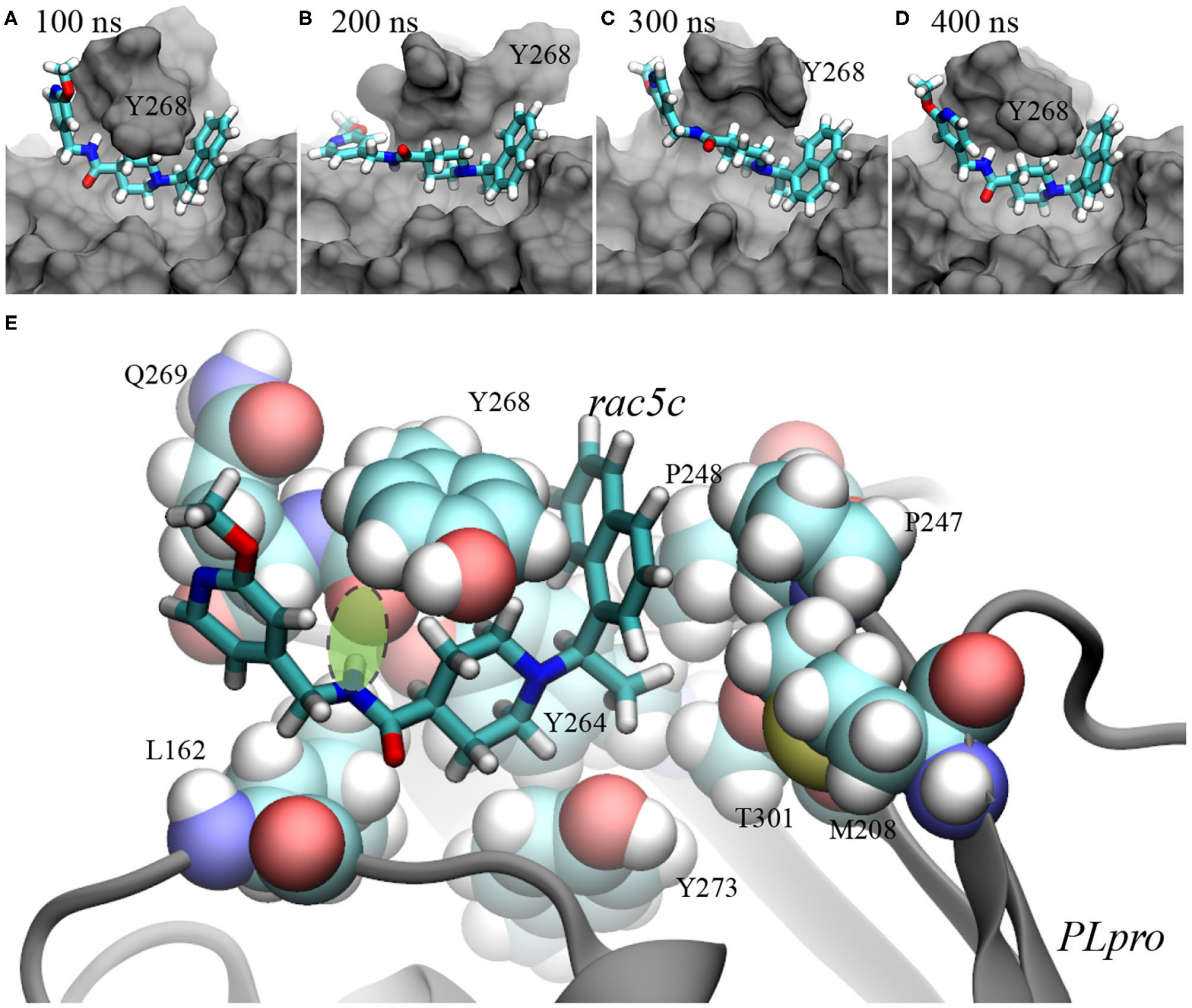
Illustration of rac5c's conformations inside the substrate-binding site of PLpro. **(A–D)** rac5c's conformations in the MD trajectory, at 100 ns **(A)**, 200 ns **(B)**, 300 ns **(C)**, and 400 ns **(D)**. rac5c is in the stick representation and PLpro is in the molecular surface representation. We provided pdb files for these complex conformations in the Supplementary Materials. **(E)** rac5c's coordination with nearby PLpro's residues. The key hydrogen bond is highlighted with a light-green oval.

From the simulation trajectory, we observed an interesting dynamic interaction between rac5c and PLpro, especially the β-turn (including residues from 264 to 271 with the sequence YTGNYQCG) as shown in Figure 2C. At the beginning (Figure 2D), Y268 of PLpro (modeled from the crystal structure with the PDB entry 6WX4) was in an “up” conformation solvated in water. Within 100 ns, Figure 4A shows that Y268 moved into a new conformation (the “down” conformation) surrounded by a U-shaped rac5c molecule, which highlights the convergence into a complex structure similar to that of the rac3j-PLpro (SARS-CoV) complex, as shown in Supplementary Figure 3. However, the local structure was still not optimized and Y268 rearranged itself back into water at about 200 ns (Figure 4B). The pyridine fragment (with the -OCH3 group) in rac5c was flexible in the PLpro's pocket. Note that the similar fragment in rac3j was partly stabilized by a co-crystallization agent DMSO (Supplementary Figure 3). Meanwhile, the other fragment naphthalene in rac5c was pinned at its original position, preventing the escape of rac5c from the pocket.

At 300 ns, Y268 moved into contact with rac5c again and was also stacked with the neighboring residue Q269 (Figure 4C). Consequently, interaction energies between rac5c and PLpro temporarily increased (Figure 3C), indicating a weaker binding. After that, Y268 quickly moved into a full contact with rac5c again (Figures 3C, 4D) and remained at that pose for the rest of MD simulation. For comparison, interaction energies near the end of simulation were more negative (by a few kcal/mol) than those near the beginning (Figure 3C), suggesting that the rac5c-PLpro complex was structurally improved/optimized during the MD simulation.

The molecular mechanism of binding between rac5c and PLpro is illustrated in Figure 4E. First, the naphthalene fragment along with an attached methyl group is highly hydrophobic and is favorably surrounded by Y268, P248, P247, T301 and M208 (Figure 4E), which accounts for its stability during the entire MD simulation (Figures 4A–D). Second, the piperidine fragment in the middle of rac5c also interacts hydrophobically with surrounding Y273, Y264, and Y268. Finally, the pyridine fragment (with attached -OCH3 group) only interacts weakly with nearby alkyl-group-containing side-chains of Q269 and L162, and thus is flexible. Additionally, the peptide-backbone fragment forms a hydrogen bond with the oxygen atom in the backbone of Y268, further stabilizing Y268 in its energetically favorable “down” conformation. All these atomic coordination's allowed rac5c to be bound stably inside the PLpro's pocket.

## 3. Conclusions

In summary, leveraging our powerful HPC cluster, we applied the flexible docking approach to screen the entire FDA-approved drug library for drug candidates that can inhibit PLpro. Due to the urgency of the pandemic, drug repurposing might provide an immediate and temporary solution with already known safety and PK profiles of each approved drug. Unfortunately, both our *in silico* and previous *in vitro* (Klemm et al., 2020) studies suggest that drug repurposing might not be a viable way to discover efficacious inhibitors for PLpro. We further studied rac5c which is known to inhibit PLpros of both SARS-CoV and SARS-CoV-2. Without crystal structures for the rac5c-PLpro complex, the molecular mechanism of rac5c's binding in the PLpro's pocket was not well-understood. Through a long (~450) MD simulation, we confirmed that rac5c can be stably bound inside the PLpro's pocket, which prevents PLpro from performing its biological function of binding and processing its protein targets. From the atomic coordination's between rac5c and PLpro that was revealed from MD simulation, we found that in rac5c only the pyridine fragment (with attached -OCH3 group) was bound loosely in the pocket, while the remaining part provides essential interactions (both hydrophobic and hydrogen-bond) with PLpro. Thus, it is feasible to optimize the pyridine fragment as well as the attached functional group to enhance the binding affinity.

Supplementary to existing experimental studies for PLpro, our *in silico* investigation provided invaluable insights for drug discovery, from the point of view of atomic-level structures. With collaborative *in silico* and *in vitro*/*in vivo* efforts, it can be expected to accelerate the discovery of a drug candidate targeting PLpro of SARS-CoV-2.

## 4. Methods

### 4.1. MD Simulations

MD simulations were performed to investigate the molecular insight and binding stability for ergotamine and rac5c in the active site of PLpro. The crystal structure of SARS-CoV-2's PLpro (Figure 1A) was obtained from the Protein Data Bank (PDB) entry 6WX4. The pdb file of ergotamine (Drugbank accession number DB00696) was converted from the mol2 file downloaded from ZINC 15 database (ZINC ID ZINC000052955754) (Sterling and Irwin, 2015). To get the pdb file for rac5c, we modified the rac3c's pdb file obtained from the crystal structure (PDB entry 4OVZ). We employed the NAMD 2.13 package (Phillips et al., 2005) running on an IBM Power cluster for the MD simulations of PLpro with or without a bound ligand. All systems were solvated in 107 × 107 × 107 Å^3^ water (TIP3P model, Jorgensen et al., 1983; Neria et al., 1996) boxes with the NaCl concentration 0.15 M, and the periodic boundary conditions (PDB) were applied in all three dimensions. The CHARMM36 force field (MacKerell et al., 1998) was used for PLpro whereas the standard force field was used for the ions (Beglov and Roux, 1994). As for ergotamine and rac5c, we generated their corresponding force fields using the SwissParam tool (Zoete et al., 2011). The electrostatic interactions were calculated with the particle-mesh Ewald (PME) method with the grid size of 1 Å set in all dimensions, while the van der Waals (VDW) interactions were handled with a smooth cutoff distance of 10–12 Å. We applied the Langevin thermostat (Allen and Tildesley, 1987) to maintain the temperature T at 300 K as well as the Nosé-Hoover method (Martinetz and Schulten, 1994) to keep the pressure constant at 1 bar. All production runs were carried out in the NVT ensemble with the simulation time-step set to 2 fs (rigid bonds for all).

### 4.2. Docking Simulations

We used AutoDock Vina (Trott and Olson, 2010), the most commonly used open-source software tool, to carry out the molecular docking simulations. In addition to rigid docking, we also used the flexible docking option which allows limited flexibility of selected receptor side-chains aiming to reflect a more realistic ligand-protein interaction environment under reasonable computer processing time. The set of residues allowed to move in the flexible docking simulation includes: L162, D164, R166, M208, S245, Y264, Y268, Y273, T301, and D302. These mobile residues in the binding pocket were selected due to their proximity to the tested ligands. The search box size was set to 22 × 14 × 32 Å, which includes the entire active site of PLpro (see *inset* in Figure 1B). We set the exhaustiveness and num_modes to be 8 and 1,000, respectively. To prepare the ligands and target protein for the docking simulations, we used the scripts provided with the AutoDock Tools (Morris et al., 2009) suite such as prepare_ligand4.py, prepare_receptor4.py and prepare_flexreceptor4.py to generate the corresponding input files required by AutoDock Vina. The list of small molecules (FDA-approved drugs) used in this study was downloaded from DrugBank (https://zinc15.docking.org/catalogs/dbfda) which contains 1,426 different compounds. Including different protonation states, totally we have 2,173 variants of mol2 files.

## Data Availability Statement

The original contributions presented in the study are included in the article/Supplementary Material, further inquiries can be directed to the corresponding author/s.

## Author Contributions

BL designed the project. TH and BL carried out all docking and MD simulations, respectively. TH, WC and BL discussed the results. TH and BL wrote paper together with inputs from WC. All authors contributed to the article and approved the submitted version.

## Conflict of Interest

TH, WC, and BL were employed by IBM.

## Supplementary Material

MD simulation of apo PLpro; results from the rigid docking approach; crystal contacts (PDB entry: 4OVZ) that may affect the binding pose of rac3j in PLpro of SARS-CoV; movies showing Zn^2+^ dynamics in the fingers domain (Supplementary Video 2), ergotamine in the PLpro's pocket (PLpro-ergotamine.mpg) and rac5c in the PLpro's pocket (PLpro-rac5c.mpg); pdb files for the complex of rac5c and PLpro at various simulation times shown in Figure 4.
