# Supplementary material for: In silico Exploration of Inhibitors for SARS-CoV-2's Papain-Like Protease
Source: Front Chem. 2021 Feb 4;8:624163. doi: 10.3389/fchem.2020.624163 (PMC7889802; doi:10.3389/fchem.2020.624163)
Supplement: Supplementary file 1 [file Data_Sheet_1.PDF]

In Silico Exploration of Efficacious Inhibitors for SARS-CoV-2's Papain-like Protease

Tien Huynh, Wendy Cornell and Binquan Luan*

Computational Biological Center, IBM Thomas J. Watson Research, Yorktown Heights, New York 10598, USA

E-mail: bluan@us.ibm.com

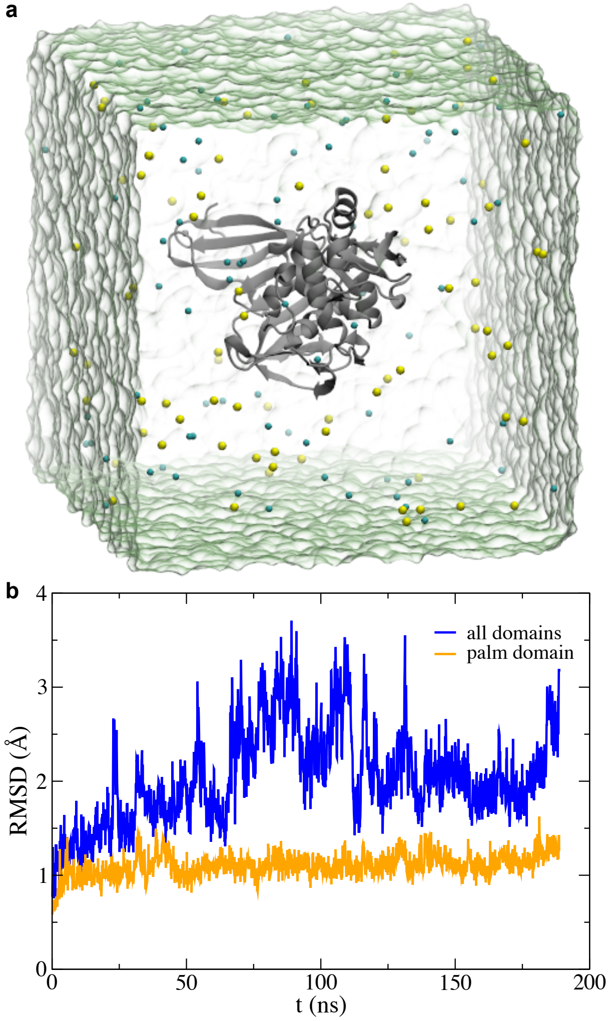

Figure S1: MD simulation of apo-PLpro. a) Simulation system. PLpro is in the cartoon representation; Na^+ (yellow) and Cl^- (cyan) are shown as van der Waals spheres; water is shown transparently. b) RMSDs of the backbone in the entire protein (blue) and RMSDs of the backbone in the palm domain only (orange).

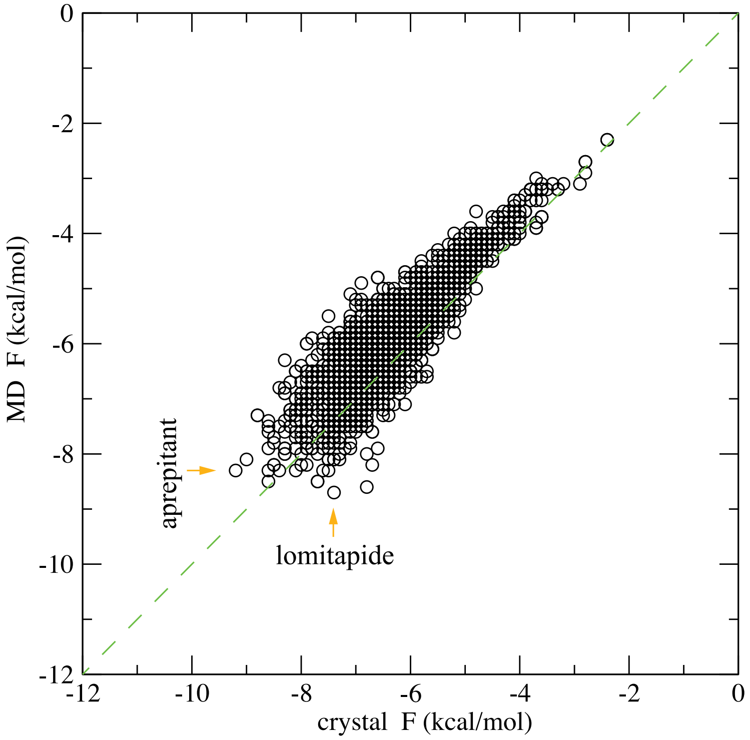

Figure S2: A scatter plot showing results from the rigid docking simulations for the MD-equilibrated PLpro and crystal-structure PLpro.

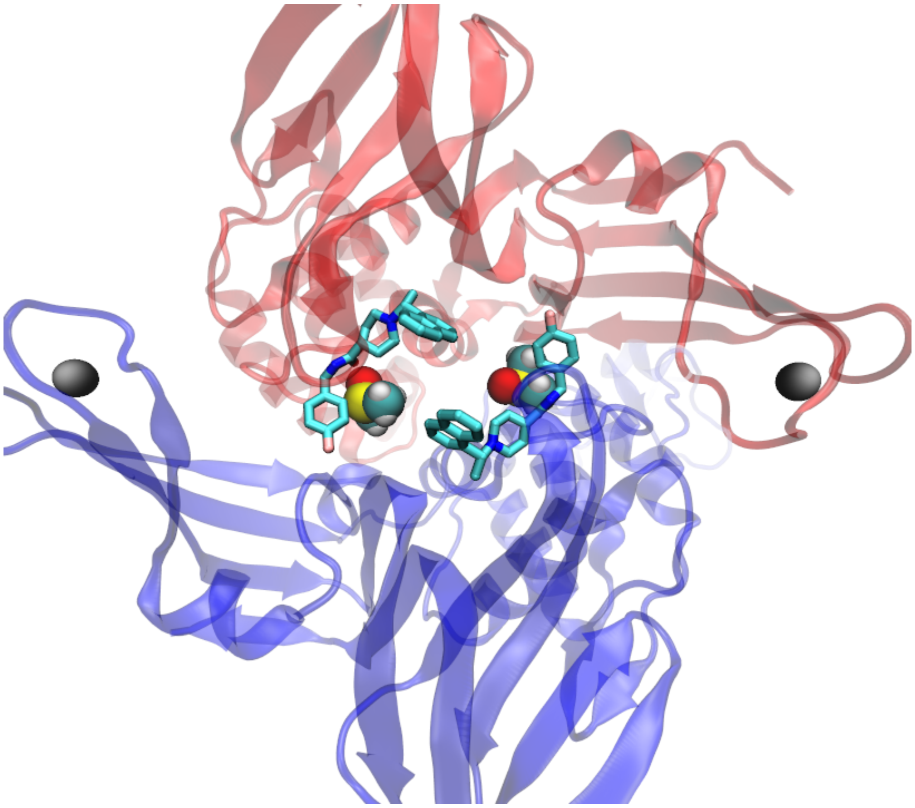

Figure S3: Illustration of the PLpro dimers with bound rac3j and DMSO molecules in the crystal structure (PDB entry: 4OVZ). two PLpros (red and blue) are in the cartoon representation; DMSO molecules are in the van der Waals representation; rac3j molecules are in the stick representation; two Zn^{2+} ions in their respective fingers domain are shown as gray spheres.
